# A cross-country comparison of the prevalence of exposure to tobacco advertisements among adolescents aged 13–15 years in 20 low and middle income countries

**DOI:** 10.1186/1617-9625-11-11

**Published:** 2013-05-23

**Authors:** Israel T Agaku, Akinyele O Adisa, Akindayo O Akinyamoju, Samuel O Agboola

**Affiliations:** 1 Africa Tobacco Control Regional Initiative, Plot 397B, George Crescent, Agbalajobi Estate, Off Wempco Road, Lagos, Ogba-Ikeja, Nigeria; 2 Department of Oral Pathology, College of Medicine, University of Ibadan, Ibadan, Nigeria; 3 Department of Oral and Maxillofacial Surgery, University of Ibadan, Ibadan, Nigeria

**Keywords:** Advertisements, Tobacco promotion, Susceptibility, Smoking, Adolescents, Cigarette, Addiction, Developing countries

## Abstract

**Background:**

This study assessed the prevalence and influence of exposure to pro-tobacco advertisements among adolescents in 20 low and middle income countries (LMICs).

**Methods:**

The 2007–2008 Global Youth Tobacco Survey was analyzed for students aged 13–15 years in 20 LMICs. Overall and sex-specific prevalence of exposure to tobacco advertisements in several media, as well as the prevalence of smoking susceptibility (i.e., the lack of a firm commitment among never smokers not to smoke in the future or if offered a cigarette by a friend) were assessed. The variability of the point estimates was assessed using 95% confidence intervals (CI). Logistic regression was used to assess the effect of exposure to multiple (i.e., ≥2) pro-tobacco advertisements on current smoking, adjusting for age and sex (*P* < 0.05). Data were weighted and analyzed with Stata version 11.

**Results:**

Overall country-specific prevalence for different advertisement sources ranged as follows: movies/videos (78.4% in Lesotho to 97.8% in Belize); television programs (48.7% in Togo to 91.7% in the Philippines); newspapers/magazines (29.5% in Togo to 89.7% in the Philippines); and outdoor community events (30.6% in Rwanda to 79.4% in the Philippines). The overall proportion of never smokers who were susceptible to cigarette smoking ranged from 3.7% in Sri Lanka to 70.1% in Kyrgyzstan. Exposure to ≥2 sources of pro-tobacco advertisements was associated with significantly increased odds of cigarette smoking among adolescents in several countries including South Africa (adjusted odds ratio, *a*OR = 4.11; 95% CI:2.26-7.47), Togo (*a*OR = 3.77; 95% CI:1.27-11.21), the Former Yugoslav Republic of Macedonia (*a*OR = 1.42; 95% CI:1.01-1.99), Republic of Moldova (*a*OR = 1.53; 95% CI:1.11-2.12), Belize (*a*OR = 13.95; 95% CI:1.91-102.02), Panama (*a*OR = 5.14; 95% CI: 2.37-11.14) and Mongolia (*a*OR = 1.52; 95% CI:1.19-1.94).

**Conclusion:**

Prevalence of exposure to various pro-tobacco advertisements was high among adolescents in the LMICs surveyed. Enhanced and sustained national efforts are needed to reduce exposure to all forms of tobacco advertising and promotional activities.

## Introduction

As declines in cigarette smoking occur in developed nations in recent years, the tobacco industry has increasingly targeted low and middle income countries (LMICs) in order to increase sales and profits [1]. During 2005 and 2010, the volume of sales of cigarettes declined by 12% in Western Europe and 20% in North America, while increasing by 17% in the Middle East region and North Africa combined [2]. Along with Asia-Pacific, the Middle East and Africa have continued to see growths in cigarette volume sales at a rate higher than the rest of the world [2-4]. The Euromonitor International- a global market research report- has projected a 7% growth in the number of smokers in the Middle East and Africa between 2010 and 2015- the fastest growth rate of smokers in the world [5]. Changes in demographics (favoring a growth in the young adult population), coupled with relatively lax tobacco control policies and lower levels of health awareness, have all contributed to the boost in volume sales of cigarettes observed in LMICs in recent years [2]. In addition, the tobacco industry has established a strong presence in several LMICs, as indicated by the construction of multi-million dollar tobacco plants in several countries including Nigeria, Senegal, and South-Africa, among others [6-8]. More so, political unrest in several LMICs including Egypt, Libya, Tunisia, Yemen, and Bahrain, may have contributed to a boost in illicit trade of tobacco products [2].

The susceptibility of youths to social influences to use tobacco products has made them an important target of the tobacco industry [9]. Research has suggested that certain tobacco companies developed a global brand through strategic advertising and marketing plans that targeted youths by highlighting themes designed to appeal to young people such as independence, hedonism, freedom, and comfort [10]. The industry has also used corporate social responsibility tactics to improve its corporate image [11], and promote tobacco products among youths. In addition, the increased expenditures on internet advertising of tobacco products by major cigarette manufacturers in recent years [12] may likely have an influence on adolescents in LMICs due to the global reach of such internet marketing activities. Such promotion of tobacco products to youths undermines national and international efforts to protect children and youths from the dangers of tobacco use [13]. The United States’ Surgeon General’s report has concluded that such tobacco promotional activities have a causal effect on smoking initiation among young people [9].

Despite these developments, only very little information exists on the reach and effect of pro-tobacco advertising among youths in developing countries [14]. To fill this gap in knowledge, this study assessed the prevalence and correlates of pro-tobacco advertisements among adolescents aged 13–15 years in 20 LMICs, as well as the prevalence of smoking susceptibility among adolescents that had never smoked cigarettes in these countries, using data from the Global Youth Tobacco Survey.

## Methods

### Data source

#### *Global youth tobacco survey (GYTS)*

The GYTS is a school-based survey that was initiated by the World Health Organization (WHO); the United States Centers for Disease Control and Prevention, and the Canadian Public Health Association. The survey collects tobacco use data at four-year intervals from students aged 13–15 years using a two stage cluster sample. Schools are selected with probability proportional to school enrolment size during the first stage, and then classes within participating schools are selected as a systematic equal probability sample with a random start during the second stage. All students in the selected classes are eligible to participate in the survey. The GYTS survey methodology has been described in detail elsewhere [15].

#### *Sample*

For this analysis, we excluded all sub-national data (i.e., provincial, city or state level data) to allow direct comparisons of country-level data. Also, all countries whose most recent national GYTS data was collected prior to 2007 were excluded to allow comparisons of more recent data. In total, 2007–2008 data was analyzed for 20 LMICs (defined as countries with gross national income per capita ≤ $12,475 as of 2011) [16]. This categorization included low income, lower-middle income, and upper middle income countries [16].

The 20 countries for which data was available for the outcome measures of interest are presented in this study by geographic region based on the six WHO regions. These included: African region (*n* = 6 countries: Botswana; Lesotho; Rwanda; Seychelles; South Africa and Togo); Eastern Mediterranean region (*n* = 3 countries: Islamic Republic of Iran; Tunisia and Yemen); Europe (*n* = 5 countries: Kyrgyzstan; the Former Yugoslav Republic of Macedonia; Republic of Moldova; Montenegro; and Serbia); Region of the Americas (*n* = 2 countries: Belize; and Panama); South-East Asia (*n* = 2 countries: Myanmar and Sri Lanka) and Western Pacific region (*n* = 2 countries: Mongolia; and Philippines).

### Measures

Questionnaires were standardized and selected question items were similarly worded for all countries, thus allowing for direct cross-country comparisons.

#### *Current cigarette smoking*

Current cigarette smoking was defined as a report by a student that they had smoked cigarettes on ≥1 day during the past 30 days.

#### *Smoking susceptibility*

Respondents who had never experimented with cigarette smoking, even one or two puffs, were defined as “never smokers.” Never smokers who were susceptible to smoking were defined as those who responded in any way other than “Definitely not” to the following two questions: “at any time during the next 12 months, do you think you will smoke a cigarette?” or “if one of your best friends offered you a cigarette, would you smoke it?”

#### *Exposure to smoking incidents in movies/videos*

Exposure to smoking incidents in movies/videos was assessed using the question: “When you watch TV, videos, or movies, how often do you see actors smoking?” Respondents who answered “I never watch TV, videos, or movies” were excluded from the analysis, whereas those who answered “a lot” or “sometimes” were categorized as being exposed to smoking incidents in movies/videos. Respondents who answered “never” were categorized as being non-exposed.

#### *Exposure to pro-tobacco advertisements on television programs*

Exposure to pro-tobacco advertisements on television programs was assessed using the question: “During the past 30 days (one month), when you watched sports events or other programs on TV how often did you see cigarette brand names?” Respondents who answered “I never watch TV” were excluded from the analysis, whereas those who answered “a lot” or “sometimes” were categorized as being exposed to pro-tobacco advertisements in television programs. Respondents who answered “never” were categorized as being non-exposed.

#### *Exposure to pro-tobacco advertisements in newspapers/magazines*

Exposure to pro-tobacco advertisements in newspapers/magazines was assessed using the question: “During the past 30 days (one month), how many advertisements or promotions for cigarettes have you seen in newspapers or magazines?” Respondents who answered “A lot” or “A few” were categorized as being exposed to pro-tobacco advertisements in newspapers/magazines whereas those who answered “None” were categorized as being non-exposed.

#### *Exposure to pro-tobacco advertisements at outdoor community events*

Exposure to pro-tobacco advertisements at outdoor community events was assessed using the question: “when you go to sports events, musical concerts, community events, or cultural festivals, how often do you see advertisements for cigarettes?” Respondents who answered “I never attend sports, musical concerts, community events, or cultural festival” were excluded from the analysis, whereas those who answered “a lot” or “sometimes” were categorized as being exposed to pro-tobacco advertisements at outdoor community events. Respondents who answered “never” were categorized as being non-exposed.

#### *Socio-demographic characteristics*

Socio-demographic characteristics assessed included age (13, 14 or 15 years); and sex (boy or girl).

### Statistical analysis

The target population of the GYTS in each country was students aged 13–15 years. Hence, we restricted all analyses to students within those ages for all 20 countries to maintain an identical population regardless of country-specific school/class grading system. This enhanced comparability of national estimates.

Data for each country were weighted to reflect the initial probabilities of selection and non-response patterns, and to post-stratify the data to known sampling frame characteristics. The prevalence of exposure to various pro-tobacco advertisements, and the proportion of never smokers who were susceptible to cigarette smoking, were assessed overall for each country and also stratified by sex; 95% confidence intervals (CI) were calculated to account for the complex survey design. Pair-wise comparison of country estimates was done using a 2 sample *t*-test. All tests were 2-tailed and the level of statistical significance was set at *P* < 0.05.

In addition, for each country, the cumulative number of pro-tobacco advertisements respondents reported being exposed to was added for each student (range: 0 to 4). This was then dichotomized as exposure to ≤1 source of pro-tobacco advertisement vs. exposure to multiple sources of pro-tobacco advertisements (2 to 4). Multivariate logistic regression was used to assess the effect of exposure to multiple pro-tobacco advertisements on current smoking, adjusting for age and sex. All analyses were performed with Stata version 11 (Stata Corp. 2009. Stata Statistical Software: College Station, TX).

## Results

Total sample sizes for all students ranged from *n* = 1219 in Yemen, to *n* = 8602 in South Africa. The proportion of 13–15 year olds among the total number of students sampled ranged from 30.2% in Rwanda to 80.3% in Panama. The proportion of boys aged 13–15 years ranged from 37.5% in Lesotho to 62.5% in Yemen. Overall response rates ranged from 51.9% (Mongolia) to 96.0% (Botswana) (Table 1).

**Table 1:** Characteristics of National Surveys of adolescents in 20 low and middle income countries, 2007-2008

**Region/Country**	**Survey year**	**Total sample size, n**	**Overall response rate(%)**	**% of all respondents aged 13**–**15 years**	% composition: boys*	% composition: girls*
**African Region**						
Botswana	2008	2207	96.0	72.8	41.6	58.4
Lesotho	2008	3426	83.2	49.8	37.5	62.5
Rwanda	2008	2284	91.8	30.2	47.2	52.8
Seychelles	2007	1508	86.0	57.0	49.4	50.6
South Africa	2008	8602	77.9	46.0	42.3	57.7
Togo	2007	4262	89.9	46.9	59.6	40.4
**Eastern Mediterranean region**						
Islamic Republic of Iran	2007	1996	85.9	66.6	52.1	47.9
Tunisia	2007	2155	92.4	70.3	50.1	49.9
Yemen	2008	1219	83.5	52.7	62.5	37.5
**Europe**						
Kyrgyzstan	2008	4038	93.2	74.4	47.4	52.6
FYR Macedonia	2008	5824	90.1	73.5	50.9	49.1
Republic of Moldova	2008	4703	84.3	75.1	44.4	55.6
Montenegro	2008	5723	92.9	58.5	48.1	51.9
Serbia	2008	4727	89.4	67.4	45.4	54.6
**Region of the Americas**						
Belize	2008	1751	93.9	61.1	47.0	53.0
Panama	2008	3543	80.0	80.3	47.1	52.9
**South**-**East Asia**						
Myanmar	2007	3118	95.2	69.5	48.9	51.2
Sri Lanka	2007	1764	85.0	79.8	50.2	49.8
**Western Pacific**						
Mongolia	2007	1831	51.9	77.8	46.6	53.4
Philippines	2007	5919	80.9	56.4	44.2	55.8

**Note**: Sample sizes “n” are unweighted while percentages are weighted. *FYR* Macedonia=the Former Yugoslav Republic of Macedonia.

*Among respondents aged 13–15 years.

Movies/videos constituted the most common source of exposure to pro-tobacco influence among adolescents aged 13–15 years. The prevalence of exposure to smoking incidents in movies/videos ranged from 78.4% (Lesotho) to 97.8% (Belize) (Table 2). Prevalence of exposure to pro-tobacco advertisements on television programs ranged from 48.7% in Togo to 91.7% in the Philippines. Prevalence estimates of exposure to pro-tobacco advertisements in newspapers/magazines were generally lower compared to the electronic (i.e., movies/videos and television programs) routes of exposure, and ranged from 29.5% (Togo) to 89.7% (Philippines). Prevalence of exposure to pro-tobacco advertisements at outdoor community events ranged from 30.6% (Rwanda) to 79.4% (Sri Lanka). There was no significant difference by sex for all types of exposure to pro-tobacco advertisements in all countries surveyed.

**Table 2:** **Prevalence of exposure to pro**-**tobacco advertisements in movies**/**videos**, **television programs**, **newspapers**/**magazines and at outdoor community events among students aged 13**–**15 years in 20 low and middle income countries**, **2007**-**2008**

**Region/Country**	**Movies/Videos**			**Television programs**			**Newspapers/ Magazines**			**Outdoor community events**		
	**Overall**	**Boys**	**Girls**	**Overall**	**Boys**	**Girls**	**Overall**	**Boys**	**Girls**	**Overall**	**Boys**	**Girls**
	**% (95%CI)**	**% (95% CI)**	**% (95% CI)**	**% (95% CI)**	**% (95% CI)**	**% (95%CI)**	**% (95%CI**)	**% (95% CI)**	**% (95% CI)**	**% (95% CI)**	**% (95% CI)**	**% (95% CI)**
**African Region**												
Botswana	90.7	91.7	90.0	68.0	65.3	70.4	48.4	47.4	49.2	55.8	56.8	54.3
	(88.7-92.7)	(88.4-94.9)	(87.7-92.3)	(65.6-70.4)	(61.9-68.7)	(66.9-74.0)	(45.1-51.8)	(43.4-51.5)	(45.3-53.1)	(52.8-58.9)	(52.6-61.0)	(50.4-58.2)
Lesotho	78.4	74.7	80.3	70.8	71.0	69.5	60.2	59.9	60.3	62.4	58.1	65.0
	(70.1-86.8)	(62.5-86.9)	(73.8-86.9)	(65.8-75.8)	(64.5-77.6)	(63.8-75.1)	(55.9-64.6)	(53.4-66.5)	(54.0-66.6)	(54.2-70.5)	(49.9-66.2)	(56.2-73.8)
Rwanda	86.4	88.8	83.9	79.0	83.1	74.7	31.1	29.2	31.9	30.6	31.2	29.4
	(81.4-91.5)	(83.8-93.9)	(76.6-91.3)	(73.4-84.6)	(78.1-88.1)	(67.2-82.2)	(26.2-35.9)	(24.5-33.9)	(23.5-40.3)	(25.9-35.3)	(25.1-37.3)	(21.1-37.7)
Seychelles	96.1	94.8	97.2	68.1	65.1	71.3	49.4	47.5	50.8	63.6	57.5	69.6
	(94.0-98.1)	(91.4-98.2)	(95.5-98.9)	(63.9-72.2)	(59.3-70.9)	(64.8-77.8)	(43.3-55.4)	(40.9-54.0)	(42.4-59.3)	(58.8-68.3)	(50.8-64.1)	(64.6-74.7)
South Africa	92.7	91.9	93.2	77.2	78.3	76.5	66.8	67.7	66.1	59.7	60.1	59.4
	(90.8-94.5)	(89.3-94.6)	(91.3-95.2)	(74.9-79.5)	(75.5-81.0)	(73.5-79.5)	(63.6-70.1)	(64.1-71.4)	(62.2-70.0)	(57.0-62.3)	(56.6-63.7)	(56.3-62.6)
Togo	80.0	79.6	80.5	48.7	46.8	50.8	29.5	29.2	29.5	44.3	43.7	44.7
	(74.0-86.0)	(72.7-86.4)	(74.5-86.4)	(40.6-56.8)	(36.7-56.8)	(43.5-58.1)	(23.0-35.9)	(21.7-36.7)	(23.6-35.3)	(34.3-54.2)	(33.5-53.9)	(33.4-55.9)
**Eastern Mediterranean region**												
Islamic Republic of Iran	94.7	93.2	96.3	64.8	64.6	64.8	48.2	48.0	48.4	59.8	60.4	58.6
	(93.2-96.3)	(91.5-94.9)	(94.3-98.4)	(58.6-71.0)	(56.4-72.9)	(57.3-72.4)	(43.0-53.3)	(39.3-56.7)	(41.3-55.5)	(53.2-66.3)	(50.8-70.1)	(49.5-67.6)
Tunisia	97.6	96.8	98.3	68.3	69.5	67.0	58.7	58.4	59.0	64.4	66.8	61.4
	(96.6-98.6)	(95.6-98.0)	(97.3-99.3)	(65.1-71.5)	(65.7-73.3)	(62.7-71.4)	(55.0-62.5)	(53.9-62.9)	(53.5-64.5)	(60.3-68.5)	(63.3-70.4)	(55.6-67.2)
Yemen	90.4	90.1	91.4	77.6	79.4	73.4	62.3	64.4	57.9	62.6	61.3	64.1
	(85.8-95.0)	(83.7-96.6)	(85.0-97.8)	(72.1-83.1)	(70.8-88.0)	(61.3-85.6)	(53.2-71.3)	(53.3-75.6)	(48.8-67.0)	(52.7-72.4)	(47.6-74.9)	(51.5-76.7)
**Europe**												
Kyrgyzstan	93.4	92.4	94.4	72.7	70.7	74.5	55.2	58.9	51.9	56.7	53.1	59.8
	(91.0-95.9)	(88.3-96.5)	(92.4-96.4)	(70.4-75.0)	(67.1-74.3)	(70.7-78.4)	(51.8-58.7)	(54.9-62.9)	(47.1-56.8)	(51.5-61.8)	(46.7-59.5)	(54.8-64.8)
FYR Macedonia	92.9	92.9	92.9	71.0	70.8	71.0	65.2	63.4	67.0	57.9	58.2	57.7
	(91.3-94.5)	(91.1-94.7)	(90.8-94.9)	(68.9-73.1)	(67.8-73.8)	(68.4-73.7)	(63.2-67.2)	(61.0-65.9)	(63.9-70.1)	(55.9-60.0)	(55.8-60.5)	(54.4-61.0)
Republic of Moldova	94.1	94.0	94.4	66.6	68.4	64.9	58.1	58.6	57.7	43.6	45.2	41.9
	(93.0-95.3)	(92.6-95.5)	(93.0-95.9)	(64.3-69.0)	(65.5-71.2)	(61.3-68.5)	(55.6-60.6)	(54.1-63.1)	(53.8-61.6)	(40.2-47.1)	(41.1-49.2)	(37.9-46.0)
Montenegro	96.7	96.1	97.4	66.5	67.7	65.5	58.2	58.3	58.1	43.6	43.5	43.2
	(96.0-97.5)	(95.3-96.9)	(96.3-98.6)	(64.5-68.6)	(64.3-71.0)	(62.3-68.6)	(55.2-61.1)	(54.4-62.2)	(53.9-62.3)	(40.5-46.7)	(39.9-47.1)	(39.0-47.5)
Serbia	97.3	96.3	98.4	73.8	75.3	73.0	63.8	65.2	62.8	53.9	54.0	53.2
	(96.4-98.2)	(94.5-98.0)	(97.7-99.2)	(71.6-76.1)	(72.1-78.5)	(69.7-76.2)	(61.5-66.2)	(61.9-68.5)	(59.8-65.7)	(51.2-56.6)	(50.1-58.0)	(49.7-56.6)
**Region of the Americas**												
Belize	97.8	97.9	97.7	77.1	78.0	76.2	58.3	59.7	56.7	64.0	64.0	64.1
	(97.1-98.4)	(96.9-98.8)	(96.6-98.7)	(72.8-81.5)	(73.2-82.8)	(70.6-81.7)	(53.5-63.1)	(53.8-65.6)	(51.5-62.0)	(59.0-69.0)	(58.0-70.1)	(58.1-70.0)
Panama	87.1	87.6	86.6	57.8	59.5	56.2	56.7	59.9	54.3	-- *	-- *	-- *
	(84.9-89.3)	(84.8-90.4)	(84.1-89.2)	(55.7-59.8)	(55.6-63.4)	(52.8-59.6)	(54.3-59.2)	(56.6-63.3)	(50.2-58.3)			
**South**-**East Asia**												
Myanmar	94.3	94.4	94.4	65.1	64.3	66.0	78.7	78.5	78.9	67.2	68.8	65.2
	(92.4-96.2)	(91.8-96.9)	(91.5-97.2)	(61.6-68.6)	(60.7-68.0)	(61.7-70.4)	(74.9-82.4)	(74.0-82.9)	(74.1-83.6)	(63.0-71.4)	(63.5-74.2)	(60.0-70.5)
Sri Lanka	93.6	92.7	94.5	72.8	74.5	71.2	68.4	68.8	68.0	79.0	78.3	79.6
	(91.9-95.4)	(89.6-95.7)	(92.8-96.3)	(68.7-76.9)	(69.4-79.7)	(66.7-75.7)	(64.6-72.3)	(62.4-75.1)	(63.2-72.8)	(75.5-82.5)	(73.6-82.9)	(75.1-84.2)
**Western Pacific**												
Mongolia	80.9	81	80.7	54.6	57.4	51.5	40.8	42.1	39.2	48.9	47.0	50.1
	(74.1-87.8)	(74.3-87.6)	(73.1-88.2)	(46.2-63)	(47.0-67.8)	(44.2-58.8)	(35.5-46.1)	(36.4-47.8)	(33.1-45.4)	(39.6-58.3)	(34.6-59.5)	(42.9-57.4)
Philippines	93.1	94	92.3	91.7	90.5	92.8	89.7	93.5	86.0	79.4	81.1	78.0
	(92.2-93.9)	(92.5-95.6)	(91–93.6)	(90.1-93.3)	(88.1-92.9)	(91.1-94.4)	(83.2-96.3)	(83.9-103)	(77.2-94.8)	(77.5-81.3)	(78.1-84.1)	(75.4-80.5)

**Note**: *CI*, confidence interval; *FYR* Macedonia, the Former Yugoslav Republic of Macedonia.

* Outcome measure not calculated because of non-availability of data (i.e., question was not asked).

The proportion of never smokers who were susceptible to cigarette smoking ranged from 3.7% in Sri Lanka to 70.1% in Kyrgyzstan (Table 3). The proportion of boys that were susceptible to cigarette smoking was significantly higher than the proportion of susceptible girls in the following countries: Botswana, Tunisia, Panama and Myanmar. In all other countries however, there was no significant gender difference in the prevalence of smoking susceptibility among never smokers.

**Table 3:** Proportion of never smokers aged 13–15 years in 20 low and middle income countries who were susceptible to cigarette smoking, 2007-2008

**Region/Country**	**Overall proportion of never smokers who were susceptible to cigarette smoking,% (95% CI)**	**Proportion of male never smokers who were susceptible to cigarette smoking,% (95% CI)**	**Proportion of female never smokers who were susceptible to cigarette smoking,% (95% CI)**
**African Region**			
Botswana	27.1 (22.5-31.7)	33.3 (27.9-38.7)	22.5 (17.6-27.4)
Lesotho	33.7 (27.0-40.3)	33.7 (24.9-42.5)	33.1 (26.0-40.1)
Madagascar	12.5 (8.1-17.0)	12.3 (6.1-18.5)	12.5 (7.0-18.1)
Rwanda	10.0 (7.1-12.9)	12 (8.1-15.9)	7.8 (4.8-10.8)
Seychelles	15.4 (11.7-19.0)	14.4 (8.6-20.2)	16.2 (12.0-20.4)
South Africa	15.4 (13.2-17.6)	17.4 (14.3-20.5)	14.3 (11.6-16.9)
Togo	9.2 (6.5-12.0)	9.7 (6.1-13.3)	8.5 (5.8-11.2)
**Eastern Mediterranean region**			
Islamic Republic of Iran	8.7 (6.9-10.5)	10.3 (7.6-13.0)	7.0 (4.6-9.4)
Tunisia	19.9 (16.2-23.7)	26.7 (21.6-31.8)	15.5 (11.3-19.8)
Yemen	24.1 (18.8-29.4)	22.1 (13.4-30.9)	27.4 (20.5-34.2)
**Europe**			
Kyrgyzstan	70.1 (64.9-75.3)	64.6 (57.3-72.0)	74.2 (68.4-80.0)
FYR Macedonia	16.7 (14.9-18.4)	15.4 (13.3-17.6)	17.9 (15.4-20.4)
Republic of Moldova	18.7 (15.6-21.8)	19.7 (15.2-24.1)	18.1 (14.4-21.8)
Montenegro	16.0 (14.0-18.1)	15.7 (12.0-19.5)	16.5 (13.7-19.2)
Serbia	19.0 (16.7-21.3)	16.2 (12.5-20.0)	20.9 (17.9-23.8)
**Region of the Americas**			
Belize	21.3 (18.5-24.2)	25.1 (20.3-29.9)	18.8 (14.1-23.4)
Panama	10.0 (8.7-11.3)	12.3 (10.5-14.1)	8.3 (6.3-10.2)
**South**-**East Asia**			
Myanmar	11.4 (9.3-13.6)	15.9 (12.1-19.6)	8.1 (5.9-10.3)
Sri Lanka	3.7 (2.1-5.3)	5.2 (2.5-7.9)	2.2 (0.8-3.7)
**Western Pacific**			
Mongolia	8.1 (5.5-10.8)	8.8 (4.8-12.8)	7.8 (3.8-11.8)
Philippines	12.9 (11.0-14.9)	15.0 (11.7-18.4)	11.6 (9.4-13.7)

**Note**: *CI*, confidence interval; *FYR* Macedonia, the Former Yugoslav Republic of Macedonia.

After adjusting for age and sex, exposure to ≥2 sources of pro-tobacco advertisements was associated with significantly increased odds of cigarette smoking among adolescents in several countries including South Africa (adjusted odds ratio, *a*OR = 4.11; 95% CI:2.26-7.47); Togo (*a*OR = 3.77; 95% CI:1.27-11.21); the Former Yugoslav Republic of Macedonia (*a*OR = 1.42; 95% CI: 1.01-1.99); Republic of Moldova (*a*OR = 1.53; 95% CI: 1.11-2.12); Belize (*a*OR = 13.95; 95% CI: 1.91-102.02); Panama (*a*OR = 5.14; 95% CI: 2.37-11.14); and Mongolia (*a*OR = 1.52; 95% CI: 1.19-1.94) (Table 4).

**Table 4:** Effect of exposure to multiple sources of tobacco advertisements on current smoking among adolescents aged 13–15 years in 20 low and middle income countries, 2007-2008

**Region/Country**	**Unadjusted effect of exposure to multiple pro-tobacco advertisements on current cigarette smoking, cOR (95% CI)**	Adjusted effect of exposure to multiple pro-tobacco advertisements on current cigarette smoking, aOR (95% CI)*
**African Region**		
Botswana	1.14 (0.73-1.80)	1.39 (0.80-2.42)
Lesotho	10.01 (3.10-32.38)	6.68 (0.97-46.26)
Madagascar	2.89 (1.05-7.96)	2.82 (0.70-11.42)
Rwanda	3.52 (1.16-10.74)	2.97 (0.95-9.29)
Seychelles	1.40 (0.87-2.26)	1.63 (0.98-2.71)
South Africa	2.61 (1.59-4.28)	4.11 (2.26-7.47) ^†^
Togo	2.88 (1.51-5.48)	3.77 (1.27-11.21) ^†^
**Eastern Mediterranean region**		
Islamic Republic of Iran	0.86 (0.40-1.82)	1.28 (0.61-2.68)
Tunisia	1.31 (0.70-2.46)	2.12 (0.87-5.15)
Yemen	1.47 (0.88-2.45)	1.70 (0.74-3.90)
**Europe**		
Kyrgyzstan	0.65 (0.28-1.52)	1.09 (0.37-3.20)
FYR Macedonia	1.44 (1.07-1.94)	1.42 (1.01-1.99) ^†^
Republic of Moldova	1.65 (1.23-2.22)	1.53 (1.11-2.12) ^†^
Montenegro	0.96 (0.60-1.56)	0.89 (0.49-1.60)
Serbia	1.93 (1.12-3.33)	1.56 (0.79-3.10)
**Region of the Americas**		
Belize	1.67 (0.88-3.16)	13.95 (1.91-102.02) ^†^
Panama	3.76 (1.88-7.53)	5.14 (2.37-11.14) ^†^
**South**-**East Asia**		
Myanmar	3.76 (1.88-7.53)	4.13 (0.86-19.94)
Sri Lanka	2.30 (0.98-5.40)	2.29 (0.75-6.98)
**Western Pacific**		
Mongolia	2.70 (1.11-6.52)	1.52 (1.19-1.94) ^†^
Philippines	1.02 (0.95-1.08)	1.01 (0.91-1.12)

**Note**: Referent category=exposure to ≤1 source of pro-tobacco advertisements; *cOR*, crude odds ratios; *aOR*, adjusted odds ratios; *CI*, confidence interval; *FYR* Macedonia, the Former Yugoslav Republic of Macedonia.

*Adjusted for age and sex.

^†^ Statistically significant at the *P* < 0.05 level.

## Discussion

This study showed that the overall prevalence of exposure to pro-tobacco advertisements from several routes (including movies/videos, television programs, newspapers/magazines and outdoor community events) among adolescents aged 13–15 years was high in all the countries surveyed. Considering that all the 20 LMICs countries assessed in this study are parties to the WHO Framework Convention on Tobacco Control [17], which encourages parties to undertake a comprehensive ban of all tobacco advertising and promotion activities (Article 13) [13], these findings indicate that there is need for stronger implementation of domestic policies in individual countries to reduce the impact of pro-tobacco social influences.

Smoking by children and adolescents and the targeting of tobacco promotional activities to this demographic group is a fundamental issue in tobacco control. Our study showed that exposure to multiple sources of pro-tobacco advertisements was associated with smoking behavior among adolescents in several LMICs. In addition, the prevalence of smoking susceptibility among never smokers was >15% in 12 out of the 20 countries assessed. This underscores the need for policies to reduce youth exposure to pro-tobacco advertisements from all sources.

The WHO and several public health and health professional organization have recommended classifying movies with tobacco incidents as R rating, with the exception of those that portray a historical figure who smoked and those that portray the negative effects of tobacco use [18-20]. Adopting this policy may further reduce exposure to tobacco incidents in youth-rated movies. This may however not completely eliminate youth exposure to smoking incidents in movies because some youths may still view some R-rated movies [19]. Hence, other interventions that have been recommended include requiring strong anti-tobacco advertisements preceding movies that depict smoking, not allowing cigarette brand displays in movies, and requiring producers of such movies to certify that no persons or company associated with the production received any consideration for that depiction [18,20].

While the WHO Framework Convention on Tobacco Control encourages the banning of all tobacco product advertising, specific national legislation is required [13]. Very few countries have implemented 100% ban on tobacco advertisements without exemptions. Point-of-purchase advertising is the commonest exemption in countries that have implemented policies restricting tobacco advertisements [21], as is the case in countries such as Serbia, South Africa, Philippines, Pakistan, the Former Yugoslav Republic of Macedonia, Costa Rica, Cameroon and Algeria [6]. In Indonesia, even though very few restrictions exist on cigarette advertisements, local manufacturers still concentrate their efforts on point-of-purchase advertising as it is considered a low investment, high impact, and 24-hour advertisement [6].

Notably, some countries have implemented partial restrictions on point-of-purchase advertisements, such as those delimiting the distance of outlets with such advertisements from a school, or restricting where such point-of-purchase advertisements may be placed within outlets. For example, legislation in South Africa restricts point-of-sale displays to within one meter of the cash register [6]. The WHO however favors complete restrictions [13] which involves putting all tobacco products out of view, as well as complete removal of other non-tobacco items (including signs, decals, mouse pads, till covers) containing tobacco product brand name, logo, motto, selling message, or recognizable color/pattern of colors.

Stronger enforcement of policies restricting tobacco advertisements in youth-oriented magazines as well as in newspapers may help reduce the prevalence of exposure to pro-tobacco advertisements through these media. Granted, this may not completely eliminate youth exposure to pro-tobacco advertisements in newspapers/magazines since some youths may still be exposed to such advertisements in international newspapers/magazines in print form or over the internet. However, if such restrictions are coupled with population based strategies that denormalize tobacco use, such as hard-hitting media campaigns that warn about the dangers of tobacco use, this may help prevent smoking initiation among never smokers, and prevent relapse among youths that have quit smoking. In addition, this may encourage youths that currently smoke to quit [22].

In 2007, the Nigerian federal government and a few state governments initiated lawsuits against three major tobacco companies: British American Tobacco, Phillip Morris and International Tobacco, for allegedly targeting under-aged minors with tobacco products [23]. While there has been no resolution to these cases to date, this development is however interesting as this is the first litigation between a developing country and the tobacco industry. How this case will affect tobacco promotional activities in Nigeria and other LMICs remains to be seen. Nonetheless, considering the heavy toll that smoking and secondhand smoke-attributable diseases place on already stretched health resources in developing countries [24-26], it is important for national governments in LMICs to invest in evidence-based strategies that will reduce tobacco use among youths, and avert excess death and disease.

### Strengths and limitations

This study compared exposure to pro-tobacco advertisements among adolescents in several LMICs. Nationally representative data from students aged 13–15 years old from 20 LMICs in all six WHO geographic regions was assessed for the period 2007–2008. However, these findings are subject to at least five limitations. First, exposure to pro-tobacco advertisements was self-reported, and may have been subject to recall bias. Second, these data apply only to adolescents that attend school. However, in most of the countries assessed, the vast majority of adolescents aged 13–15 years are enrolled in traditional school, and thus the results may be representative of most of the students in this age bracket in the respective countries [15]. Third, the heterogeneity in school grading/class systems in the different countries made it impossible to meaningfully break down the analysis by school or grade level. Fourth, these findings may not be generalizable to respective regions as there were limited countries for which data was available. Finally, more recent data was not available because of the 4-year cycle of the GYTS.

## Conclusion

This study demonstrated that the overall prevalence of exposure to pro-tobacco advertisements from different sources including movies, television programs, newspapers/magazines and outdoor events was high among adolescents aged 13–15 years in all 20 LMICs surveyed. In addition, exposure to multiple sources of pro-tobacco advertisements was associated with smoking behavior among adolescents in several LMICs. Enhanced and sustained national efforts in LMICs are needed to reduce exposure to all forms of tobacco advertising and promotional activities.

## Competing interests

The authors have no competing interests to report.

## Authors’ contributions

ITA initiated the study design, analyzed the data and prepared the initial draft of the manuscript. AOA, AOA, and SOA critically reviewed and revised the manuscript. All authors read and approved the final manuscript.
